# The influence of prior brief occlusion therapy on the outcome of later amblyopia treatment in cats

**DOI:** 10.3389/fnins.2026.1772221

**Published:** 2026-02-18

**Authors:** Donald E. Mitchell, Seth Smith, Nicholas D. Murphy, Lan T. J. Dang, Nathan A. Crowder, Kevin R. Duffy

**Affiliations:** Department of Psychology & Neuroscience, Dalhousie University, Halifax, NS, Canada

**Keywords:** acuity, amblyopia, animal model, monocular deprivation, patching, vision, visual cortex

## Abstract

**Background:**

Certain multi-centered randomized clinical trials of conventional treatment of children with amblyopia revealed greater improvement of the visual acuity of the amblyopic eye in patients that were never treated compared to those with a prior history of unsuccessful therapy. As a step toward a possible explanation for this phenomenon, this study investigated the influence of early prior brief treatment of amblyopia on the success of later treatment.

**Methods:**

These experiments were conducted using the well documented feline model of deprivation amblyopia. Behavioral visual acuity thresholds were made by use of a jumping stand and a two-alternative forced choice procedure.

**Results:**

Six amblyopic kittens that received a very brief period of occlusion of the non-amblyopic eye prior to a much longer subsequent period of such occlusion exhibited less recovery of the visual acuity of the amblyopic eye (group mean 1.44 c/deg) compared to 7 control amblyopic animals that received only the second period of treatment (group mean 2.57 c/deg).

**Conclusion:**

The results from this animal model indicate that prior visual history may impact the capacity for neural plasticity and thus the potential for recovery from amblyopia. From a clinical standpoint the data suggest adoption of a 3-E protocol to optimize the efficiency of occlusion therapy for amblyopia in which occlusion is initiated Early, applied in an Exact manner, and be Enduring in length.

## 1 Introduction

Human amblyopia has been modelled in animal species for more than 60 years beginning with studies on kittens (Wiesel and Hubel, 1963, 1965) and monkeys (von Noorden et al., 1970; Hubel et al., 1977; Kiorpes and Movshon 1996) and later on rodents (Drager, 1978). Of the four clinical types of human amblyopia that were classified historically according to the putative cause, namely an association with anisometropia (unequal refractive states of the two eyes), strabismus, or a combination of both, it is the fourth and least prevalent category (deprivation amblyopia) that has been modelled most frequently in research animals (Maurer and McKee, 2018; Duffy et al., 2023). Deprivation amblyopia receives its name from an association with a severe disruption of spatial imagery in one eye as can occur with an opacity of the optical media such as the crystalline lens (a cataract) or cornea. In the majority of animal studies, deprivation amblyopia is produced by surgical eyelid closure, a procedure called monocular deprivation that mimics impairments in humans but can require frequent monitoring especially in young animals as was noted in the autobiographical account of Hubel and Wiesel (2005, p. 371).

The fine-tuning of surgical procedures (e.g., Murphy and Mitchell, 1987) enabled secure and repeatable periods of monocular deprivation (MD) in kittens and monkeys so that much of what is known about experiential influences and their timing on the development of the visual pathways and of vision has been derived from study of the consequences of this form of early monocular deprivation in these two species (Mitchell and Maurer, 2022).

The last two decades has seen the emergence of multi-centered randomized clinical trials of both mainstay treatments of amblyopia such as monocular penalization with atropine or patching, as well as new therapies (e.g., Hess and Thompson, 2015) that include monocular or binocular anaglyphic video game therapy (Pedig, 2019). With one exception (Scheiman et al., 2008), the clinical trials of mainstay treatment of amblyopia by patching therapy have revealed greater improvement of the visual acuity of the amblyopic eye in children that had never been treated when compared to those with a prior history of unsuccessful treatment (Scheiman et al., 2005; Holmes et al., 2011). This observation suggests that separated episodes of treatment by patching convey benefits that are not strictly additive as the initial period of patching appeared in certain circumstances to actively reduce the efficacy of a subsequent episode of treatment. With respect to the additivity of separated episodes of treatment, experiments conducted on kitten and monkey models of respectively, deprivation (Crewther et al., 1981) and strabismic amblyopia (Wensveen et al., 2021) reveal that repeated daily episodes of normal visual exposure *can* be additive and may even restore normal visual acuity in both eyes and stereopsis. A possible explanation for the negative consequence of prior unsuccessful treatment of human amblyopia was provided by Levi and Li (2009, p. 2545) with the speculation that the first unsuccessful period of treatment might deplete a “declining pool of cortical plasticity” with age thereby reducing the efficacy of the second episode of treatment. The recent demonstration (Henneberry et al., 2023) in the cat dorsal lateral geniculate nucleus that an early period of monocular deprivation prematurely attenuates the consequences of a second instalment of such deprivation of the fellow eye provides support for this contention.

Additional data concerning possible consequences of spaced episodes of treatment were obtained several decades ago from experiments (Mitchell et al., 1984; Murphy and Mitchell, 1987) conducted on monocularly deprived kittens for which a period of reverse occlusion of certain durations was followed by a period of binocular exposure. In certain experiential circumstances the gains in visual acuity of the deprived eye that occurred during the period of occlusion of the fellow eye were not maintained afterward once vision was restored to this eye. Because the two treatments (occlusion of the non-deprived eye followed by binocular exposure) received by the kittens in these past studies were quite different, in the current pilot study we explored the consequence of application of two spaced episodes of *identical* treatment (monocular occlusion of the original non-deprived eye) but of different durations.

The design of our study was informed by results from the pioneering parametric investigation (Movshon, 1976) of the speed and extent of recovery of ocular dominance of visual cortical cells that followed a single period of reverse occlusion (RO) of various durations imposed on monocularly deprived kittens at different ages. When RO was initiated early (at 4 or 5 weeks of age), sizeable effects were evident in just 3 days and at ages as late as 7 weeks attained asymptotic values in 3 weeks or less (Movshon, 1976, Figs 3 and 17). To investigate possible interactions between separated periods of RO, we examined the impact of a short 3-day period of such treatment imposed shortly after the initial period of monocular deprivation on the results that followed a much longer episode of RO introduced at around 6 weeks of age.

## 2 Materials and methods

### 2.1 Animals

This study was conducted on 15 kittens (10 females and 5 males) from 6 consecutive litters that were born and raised in a closed breeding colony at Dalhousie University over a 2-year period that began in September 2022. All rearing and experimental procedures complied with ARRIVE standards. Experiments were conducted in accordance with protocols approved by the University Committee on Laboratory Animals at Dalhousie University that conformed to guidelines from the Canadian Council on Animal Care. Experiential rearing and visual testing occurred during the period from birth to 4 months of age during which time animals were housed in enriched colony rooms that were illuminated under a 12:12 h light/dark cycle. Prior to being weaned at around 2 months of age, kittens were housed together with the mother in a large space with open enclosures. Animals were fed dry cat chow (Loblaws, Toronto, Canada) *ad lib* and received 80 g of wet commercial cat food each day (Loblaws, Toronto, Canada; Nestle Purina, Mississauga, Canada). Behavioural tests that we employed did not require any reduction in the amount or nature of their daily food intake. Following this study, two animals remained in the colony to expand the breeding colony while all others were adopted as house pets by members of the university community after being neutered or spayed by the university veterinarian.

### 2.2 Experiential manipulations

**Figure 1:**
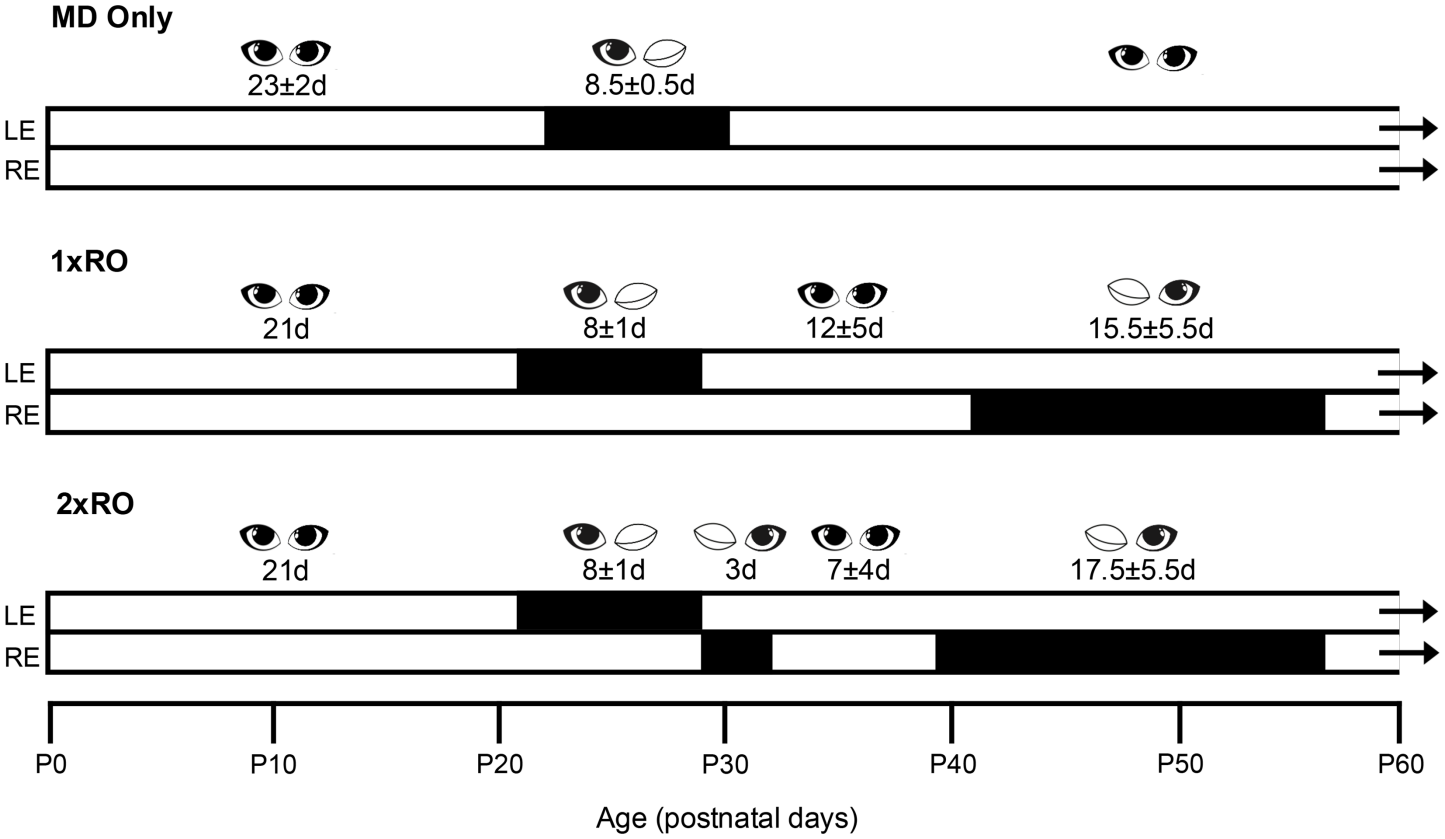
A schematic representation of the rearing history for the three groups of animals (MD only, and those that received either respectively, one (1 x RO) or two (2 x RO) periods of reverse occlusion).

Each kitten was assigned to one of three groups at around 2 weeks of age in a quasi-random order made necessary by the marked differences in litter size (from 1 to 5 kittens). The rearing history of each kitten from the six litters is listed in Tables 1, 2 together with the final acuities attained by their two eyes (Table 1). Figure 1 provides a visual aid to the rearing history of the three groups that consisted of a control group (MD only, *N* = 2 kittens) and two experimental groups that received MD followed by either 1 (1 x RO; *N* = 7) or 2 (2 x RO; *N* = 6) periods of reverse occlusion. Because all but two of the litter sizes were small it was decided to concentrate upon population of the two experimental groups, a decision made comfortable by the fact that data was available from past studies (Duffy and Mitchell, 2013; Mitchell et al., 2016) conducted in this laboratory with animals reared similarly to those in the control group. All animals received an initial 7-day period of MD by eyelid suture of the left eye from postnatal day 21 to day 28 (P21-28). With the exception of the two control animals that received no further experiential manipulation, the remaining animals received either a single (1 x RO group; *N* = 7) or else two (2 x RO group; *N* = 6) periods of reverse occlusion. One of the periods of reverse occlusion occurred at either 5 or 6 weeks of age for all animals and lasted for 2 (*N* = 2) or 3 weeks (*N* = 11). For the 7 animals assigned to the (1 x RO) group, their single period of RO occurred at this age. For the 6 kittens assigned to the other (2 x RO) group, a short 3-day period of reverse occlusion was imposed either immediately (animals PT1-2, PT4-7, PT4-8, PT6-13, and PT6-14) or 3 days after (kitten PT5-10) the initial period of MD that was terminated at about 4 weeks of age.

**Table 1:** Kitten litters, names, sex, rearing group and final acuity.

Litter	Kitten	Sex	Rearing group	Initial MD	Ages		Final acuity (c/deg)	
					RO1	RO2	Dep. Eye	Non-dep. Eye
PT1	PT1-1	F	MD + 1 x RO	P21-28	P42-54	–	3.13	6.47
	PT1-2	M	MD + 2 x RO	P21-28	P28-31	P42-54	1.87	4.95
PT2	PT2-3	M	MD + 1 x RO	P21-28	P45-56	–	2.51	6.19
PT3	PT3-4	F	MD only	P25-34	–	–	1.60	6.47
PT4	PT4-5	F	MD + 1 x RO	P21-28	P35-55	–	2.66	6.82
	PT4-6	F	MD + 1 x RO	P21-28	P35-56	–	2.34	6.47
	PT4-7	F	MD + 2 x RO	P21-28	P28-31	P35-56	0.55	6.19
	PT4-8	F	MD + 2 x RO	P21-28	P28-31	P35-56	0.70	5.00
PT5	PT5-9	F	MD + 1 x RO	P21-28	P37-58	–	1.60	6.47
	PT5-10	M	MD + 2 x RO	P21-28	P31-34	P37-58	0.78	6.19
PT6	PT6-11	M	MD + 1 x RO	P21-30	P41-61	–	2.78	6.19
	PT6-12	F	MD + 1 x RO	P21-30	P41-61	–	2.97	6.47
	PT6-13	M	MD + 2 x RO	P21-30	P30-33	P41-61	2.51	6.82
	PT6-14	F	MD + 2 x RO	P21-30	P30-33	P41-61	2.23	6.47
	PT6-15	F	MD only	P21-30	–	–	2.66	6.82

Kitten litter, individual kitten identification, sex, rearing group, age (postnatal age in days) at each experimental manipulation and final acuity for the deprived eye and the other (non-deprived) eye as measured by the binocular acuity.

**Table 2:** Kitten rearing manipulation.

Kitten	BE1	MD	BE2	RO1	BE3	RO2	BE4
MD only							
PT3-4	P0-25	P25-33	P33-				
PT6-15	P0-21	P21-30	P30-				
MD + 1 x RO							
PT1-1	P0-21	P21-28	P28-42	P42-54	P54-		
PT2-3	P0-21	P21-28	P28-45	P45-56	P56-		
PT4-5	P0-21	P21-28	P28-35	P35-55	P55-		
PT4-6	P0-21	P21-28	P28-37	P37-58	P58-		
PT5-9	P0-21	P21-28	P28-37	P37-58	P58-		
PT6-11	P0-21	P21-30	P30-41	P41-61	P61-		
PT6-12	P0-21	P21-30	P30-41	P41-61	P61-		
MD + 2 x RO							
PT1-2	P0-21	P21-28	–	P28-31	P31-42	P42-P54	P54-
PT4-7	P0-21	P21-28	–	P28-31	P31-35	P35-58	P56-
PT4-8	P0-21	P21-28	–	P28-31	P31-35	P35-56	P56-
PT5-10	P0-21	P21-28	P28-31	P31-34	P34-37	P37-58	P58-
PT6-13	P0-21	P21-30	–	P30-33	P33-41	P41-61	P61-
PT6-14	P0-21	P21-30	–	P30-33	P33-41	P41-61	P61-

The timing of the various experimental manipulations for the individual kittens. The experiential episodes are abbreviated as BE (binocular experience), MD (monocular deprivation), RO (reverse occlusion).

### 2.3 Surgical procedures

All surgical manipulations necessary for the initial monocular deprivation and for the subsequent periods of reverse occlusion were conducted by KRD or DEM. As with our customary practice (Duffy et al., 2014), animals were monocularly deprived under general gaseous anesthesia (3–4% isoflurane in oxygen) in a two-stage procedure. Preceding the surgical procedures, local anaesthesia was ensured by application of Alcaine sterile ophthalmic solution (1% proparacaine hydrochloride; CDMV Canada) to the palpebral conjunctivae. Closure of the upper with the lower palpebral conjunctivae with sterile 5–0 vicryl suture thread was followed by closure of the eyelids with 5–0 silk sutures. Upon completion of surgery, animals were administered oral Metacam (meloxicam 0.05 mg/kg) for post-procedure analgesia and they also received a topical broad-spectrum ophthalmic antibiotic (1% chloromycetin; CDMV Canada) to mitigate against post-surgical infection. The quality of eyelid closure was monitored closely on a daily basis to ensure that the eyelids remained fully closed. Termination of eyelid closure was achieved under gaseous anesthesia by removal of the conjunctival and eyelid sutures and followed the same post-surgical procedures as described for the initial eyelid closure. With the exception of 4 of 5 kittens in one litter (Litter 6) the periods of eyelid closure were observed as complete throughout their intended durations. As described in detail in the Results section, the eyelids of 4 kittens in litter 6 were observed to be open partially for a time during the intended period of occlusion.

### 2.4 Behavioral training and testing

Training and behavioural testing of grating acuity employed a jumping stand and followed procedures outlined in earlier papers (Mitchell et al., 1977; Murphy and Mitchell, 1987, Mitchell, 2013 and upgraded recently Mitchell, 2025). Visual acuity was measured longitudinally with one experimenter seated on either side of the jumping platform to minimize the emergence of a side preference and to ensure consistent behaviour across all animals over the 2 years necessary to collect the data. The method capitalizes upon a natural behaviour of kittens to descend by scrambling or jumping for a food or petting reward to one of two large adjacent square-wave grating patterns having the same period but at right-angles to each other beneath them. The positive (rewarded) stimulus was a vertical grating while the unrewarded grating was horizontal. Jumps to the latter were not rewarded and the animals were required immediately to repeat the trial. The jumping platform from which the kitten jumped was supported by two yoked laboratory jacks to enable small and continuous changes in its distance to the grating stimuli in a manner commensurate with the kitten’s ability to jump at any age. Training began on the jumping stand at 4–5 weeks of age and was usually completed in a few days.

Visual acuity for square-wave gratings were measured by a strictly descending method of limits with small decrements in stimulus magnitudes (in this case, spatial frequency) that were equated on a logarithmic scale with as many as 12 steps to an octave. In order to constrain the total number of trials the spatial frequency was increased after only one or two successful trials but, closer to threshold, the minimum number of trials was increased to five. After an error, the animal was denied the food reward and was required to repeat the trial. Once an error had been corrected, the animal was required to make five consecutively correct responses or else make seven correct responses out of a maximum of 10 trials at any spatial frequency before proceeding to the next higher spatial frequency. The threshold was defined as the highest spatial frequency for which this criterion performance level was achieved. It was typical for animals to perform flawlessly until near threshold where, in addition to errors, they would exhibit general signs of difficulty with its choice that included marked increased latency, vocalizations and variation of its patterns of gaze from the stimuli themselves to other objects in the room. The nervousness and reluctance exhibited by most kittens at the highest grating spatial frequencies prevented the use of a strict staircase procedure that incorporates trials with stimuli of progressively lower spatial frequency in the immediate aftermath of this failure. When possible, additional estimates of acuity in a single session were obtained by repetitions of a descending method of limits but starting with gratings of a different spatial frequency (usually higher) than that employed for the immediately prior assessments.

Measurement of the grating acuities of the two eyes were conducted on all kittens until they were between 3 and 4 months old at which age grating acuity as measured on the jumping stand has been shown to achieve asymptotic values (Mitchell et al., 1976). Using procedures previously described (Hogan et al., 2023), assessment of the refractive status of each eye of all 11 kittens of 3 litters (PT4, PT5, PT6) by retinoscopy at the conclusion of testing revealed that they were either emmetropic or within 0.25D of this status in each eye.

## 3 Results

As is apparent from Tables 1, 2, the litters were of very different size with two litters each of only one or two kittens while the largest two litters had either 4 or 5 kittens. For the latter two litters it was possible to assign two animals to each of the experimental exposure conditions (1 x RO and 2 x RO). The number of early experimental manipulations and their duration prevented regular longitudinal measurements of visual acuity until the kittens were at least 2 months old, a problem exacerbated by a shortage of personnel at certain times. For these reasons we concentrated upon ensuring frequent (usually daily) measurements of acuity on all animals upon completion of the periods of monocular occlusion for a period of 4–10 weeks. For every animal, the longitudinal changes of acuity were similar to those observed in the past (Mitchell et al., 1976), and interocular differences in acuity were of comparable magnitude on every testing session. As a consequence, the main focus of this study was upon the final acuity achieved by the two eyes of each animal rather than on detailed documentation of the changes that occurred longitudinally through the different episodes of visual exposure.

**Figure 2:**
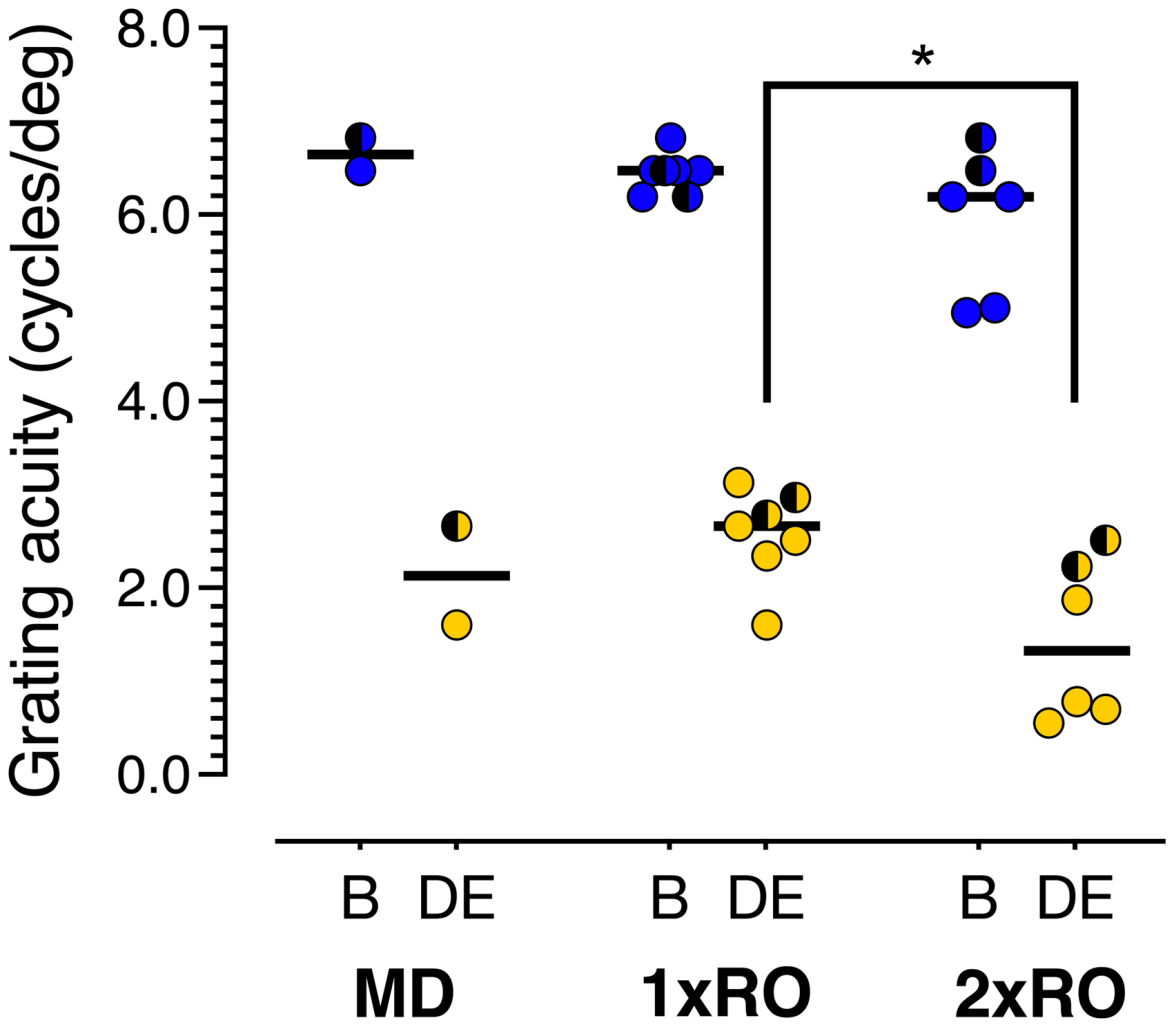
The final acuities for the deprived eye (yellow symbols) and the non-deprived eye (binocular acuity; blue symbols) of each animal in the 3 groups. The mean for each group is indicated by a short horizontal bar. The means for the 1 x 2RO and 2 x RO groups were statistically different (*p* < 0.05). The semi-shaded symbols depict results from the 5 kittens of litter PT6.

A graphical depiction of the final acuities of the two eyes of all the kittens subjected to each of the treatment regimens (1 x RO; 2 x RO; and MD control) as measured on a jumping stand when they were 3 to 4 months old are displayed in Figure 2. As mentioned in Methods, the periods of RO for four of the kittens of the last litter (PT-6) were interrupted by 2 intervals of incomplete eyelid closure that were corrected surgically within a day. In most cases the failure was with the outer stitches as the conjunctival stitches appeared to hold throughout the period of reverse occlusion. Because the visual experience obtained through the partially closed sutures was very temporary, incomplete and difficult to document, the decision was made to include the results from these animals in the study. This decision was made comfortable by the realization that the aberrant experience, albeit very temporary, could be considered an additional episode of “experiential treatment” applied approximately equally to the two experimental treatment groups. Data from the animals in the PT6 litter are depicted with half-shaded symbols in order to separate them from the results from the other litters. Consistent with our *a priori* hypothesis that multiple bouts of RO should affect vision in the deprived eye, a Student’s *t*-test (two-tailed) showed that deprived eye acuity in the 2 x RO group (Mean = 1.44, SD = 0.864) was significantly lower than the 1 x RO group (Mean = 2.57, SD = 0.504; *p* = 0.0135; Cohen’s *d* = 1.64). A sensitivity analysis revealed that this difference remained significant even if litter PT6 was excluded (*p* = 6.73 × 10^−3^; Cohen’s *d* = 2.55). This targeted comparison with the *t*-test was further supported by an omnibus analysis of grating acuity using a mixed-model ANOVA comparing vision in each eye (Deprived Eye vs. Non-Deprived Eye; within groups) and rearing conditions (MD vs. 1 x RO vs. 2 x RO; between groups). As expected, there was a significant main effect indicating acuity mediated by the Non-Deprived Eye was much higher than acuity in the Deprived Eye for all groups (*F*[1,12] = 387; *p* = 1.69 × 10^–10;^ *η^2^_G_* = 0.918). There was also a significant main effect of rearing condition (*F*[2,12] = 4.53; *p* = 0.0342; *η^2^_G_* = 0.331), which accounts for the poorer acuity of both the Deprived Eye and Non-Deprived Eye in the 2 x RO group. Finally, there was a similar drop in acuity between the non-deprived eye and deprived eye across the MD, 1 x RO, and 2 x RO rearing conditions so it was not surprising that there was no evidence of an interaction between eye and rearing condition (*F*[2,12] = 1.40; *p* = 0.283; *η*^2^_G_ = 0.0749).

## 4 Discussion

### 4.1 Additivity of experiential interventions

Although the mean visual acuity recovered by the deprived eye of animals that received two temporally separated periods of reverse occlusion was significantly lower than that for animals that received only a single period of such treatment, the size of the improvement in both groups was low. In retrospect, the outcome of this study may have been more substantial if the late (i.e., second) period of reverse occlusion had been introduced earlier in life so as to increase its potential efficacy. However, the constraints inherent with the use of cats from a laboratory breeding colony of limited size to model amblyopia, such as small and varying litter sizes and irregular pregnancies, required commitment to the original choice of experimental design until completion of the study. Nonetheless, the behavioral outcomes of this study are supported by previous electrophysiological and anatomical studies on cats and monkeys that revealed interactions between temporally adjacent experiential manipulations. In particular, these past studies reveal that an early episode of abnormal visual experience alters the effect magnitude of an interlude of similar or different anomalous visual exposure imposed at a later age (Mustari and Cynader, 1981; Faulkner et al., 2005; Smith et al., 1992; Henneberry et al., 2023).

The first of these studies was conducted on kittens in the laboratory of a past departmental colleague (Mustari and Cynader, 1981) and demonstrated that an early period of ocular misalignment (strabismus) attenuated the electrophysiological effects of an 8-day period of MD in the visual cortex imposed at P45 days as compared to that observed in response to the same deprivation imposed on normal kittens at the same age. A similar conclusion was reached 2 decades later by use of optical imaging of intrinsic signals to compare the extent of cortical territory dominated by each eye following a 10-day period of MD imposed on two groups of cats (Faulkner et al., 2005). Whereas control animals received a single 10-day period of MD at P35, animals in the experimental group had been made strabismic by myotomy of one of the extraocular muscles 3 weeks prior to imposition of the same 10-day period of MD at 35 days of age. Longitudinal measurement of the rate of ocular dominance recovery after the period of MD was found to be both slower and less complete in the animals that had been made strabismic before the period of MD. A similar finding was observed in monkeys where prior binocular dissociation via strabismic rearing reduced the efficacy of MD to produce form deprivation amblyopia (Smith et al., 1992).

That an early transitory period of MD might also produce changes in the visual cortex that influence the response to such deprivation induced much later was demonstrated in mice by the discovery that such early MD promoted an augmented response to imposition of a second period of MD imposed on the same eye (but not the fellow eye) in adulthood (Hofer et al., 2006; Hofer et al., 2009). The broader possibility that a brief early period of monocular deprivation might alter the response to a second interval of such exposure imposed shortly afterward was explored in a recent extensive anatomical and electrophysiological study conducted on kittens (Henneberry et al., 2023). Notably, it was demonstrated that an early short period of MD reduced substantially the anatomical and electrophysiological responses to a subsequent period of occlusion or retinal inactivation of the fellow eye in comparison to that observed in control animals that received only the latter deprivation.

Further insight into the consequences of temporally spaced early experiential interventions are available from studies conducted on kittens and infant monkeys that were reared with repeated *daily* episodes of typical vision followed or preceded by intervals of grossly abnormal visual exposure (Mitchell et al., 2003; Wensveen et al., 2006). These studies also provided information on the closely related issue of the extent to which the impact of separate daily episodes of visual exposure were additive. The monkeys for these studies were reared with helmets that were worn continuously from 3 to 18 weeks of age and enabled either binocular clear viewing through plano lenses in front of both eyes for part of each day followed or preceded by monocular viewing with a diffuser lens in front of one eye and a plano lens in front of the other eye. For kittens, opaque neoprene masks were used to enable the daily period of monocular visual exposure and were removed for the episode of binocular exposure. The masks required close supervision by a technician to ensure they were worn and consequently the total daily visual exposure was restricted to 7 h each day. For the remaining 17 h each day the kittens were housed in complete darkness as described in detail elsewhere (Mitchell, 2013). An additional difference between the monkey and kitten studies was in regard to the methods of behavioral testing which for the kittens permitted immediate evaluation of the visual acuity of each eye at the conclusion of the period in which they received mixed daily visual exposure. By contrast, the long behavioral training necessary for the monkeys meant that this information was obtained a year following the period of restricted daily visual exposure. Despite the substantial differences in the rearing and testing procedures for the two species, results were similar in that 2 h of daily normal visual exposure was sufficient to offset much longer periods of daily monocular exposure to allow development of normal acuity in both eyes (Mitchell et al., 2003; Wensveen et al., 2006). Importantly it was shown in kittens that two separated periods of binocular exposure were not necessarily additive and in certain circumstances one of the two binocular instalments appeared to be completely ineffective (Mitchell et al., 2006, see Fig. 5). The latter result is also consistent with the current data indicating that two separated episodes of occlusion treatment produced significantly less recovery in comparison to just one.

As summarized above, the non-additive consequences of the two temporally separated occlusion treatments administered to the amblyopic animals of our study add to a body of pre-existing data derived from animals concerning the consequences of separated episodes of selected early visual exposure. Our findings, when combined with the results of these prior studies hold important implications for clinical treatment of amblyopia in humans.

### 4.2 Suggestions for treatment of human amblyopia

Notwithstanding the reservations expressed earlier concerning the duration and spacing of the particular visual exposures employed in the present study, the results reached statistical significance. In confirmation of earlier results from research on animal models of amblyopia, it is apparent that an early or brief period of occlusion of the non-amblyopic eye may subsequently have unexpected visual consequences that may even include the emergence of bilateral amblyopia (Mitchell et al., 1984; Murphy and Mitchell, 1987). In combination, results from these animal studies suggest that in addition to existing advice that experiential treatment of amblyopia such as patching be initiated early, it should also follow an exact protocol in terms of adherence to the particular treatment and be applied for a specified time. The combined components of this treatment imply a “Three-E” protocol: Early, Exact and Enduring. In addition to the existing advice for *early* experiential intervention the specific treatment regimen should be monitored closely with respect to adherence and thus be *exact* and in addition, it should be *enduring* with respect to its duration so as to maximize the potential efficacy. Our study points to the need for perseverance with a particular protocol in order to maximize its potential effectiveness and also provides a note of caution with respect to the potential consequences of interruption of treatment.

## Data Availability

The original contributions presented in the study are included in the article/supplementary material, further inquiries can be directed to the corresponding author.
